# Education to support professional identity formation in medical students: guiding implicit social learning

**DOI:** 10.5116/ijme.63f3.ddcb

**Published:** 2023-02-28

**Authors:** Salome Scholtens, Pieter C. Barnhoorn, Joke Fleer

**Affiliations:** 1 University of Groningen, University Medical Center Groningen, Department of Health Sciences, Section Health Psychology, Groningen, the Netherlands; 2 Department of Public Health and Primary Care, Leiden University Medical Center, Leiden, the Netherlands

**Keywords:** Professional identity formation, implicit knowledge, socialisation, experiential learning, social system

## Introduction

Professional identity formation (PIF) is considered a fundamental aspect of medical education.^1^^,^^2^ It is a complex process in which a student transitions from being a lay person into a physician with full membership of the medical community of practice. While identity formation is a continuous and inevitable process that occurs within any social context during an individual's lifetime,^3^ medical students also need to develop a professional identity.^4^^,^^5^ This process is achieved through conscious and unconscious socialisation processes in which the embedded, collective knowledge of the community of practice is transferred to the learner.^4^^-^^6^ It entails the transfer of knowledge of the social context and of 'how we do things here' along with associated habits, beliefs and values to the learner.^7^^-^^10^ This process mostly occurs unconsciously, implying that much of the knowledge essential for developing a professional identity is implicit.^4^^,^^7^^,^^8^ Learners unconsciously and unintentionally 'copy' others within a shared social context, thereby acquiring collective implicit knowledge. The individuals who are emulated are often unaware of this knowledge that they transfer, which is not articulated. This implicit knowledge transfer has been termed the hidden curriculum.^10^^,^^11^

The transfer of implicit knowledge may lead to suboptimal learning outcomes and may even have adverse effects on students' wellbeing and professional behaviour.^12^^,^^13^ To counterbalance potentially suboptimal learning outcomes and to support medical students in developing the intended professional identity, medical curricula should foster students' awareness of this implicit knowledge transferral via the social context.^2^^,^^5^^,^^14^ Herein lies a challenge for the experts responsible for developing and implementing education focusing on this theme.

The establishment of a cognitive knowledge base on PIF among learners is relatively easy to implement within medical education through the transmission of explicit knowledge (e.g., via textbooks). However, the work of supporting and mediating the acquisition of implicit knowledge that shapes the students' professional identity is more difficult.^7^ The literature suggests that experiential learning and reflection guided by mentors and role models are effective strategies for influencing PIF in an educational setting.^5^^,^^11^^,^^14^ However, before learners can verbally reflect on their PIF process, they must first be taught to become aware of their socialisation process and the implicit knowledge that they have already acquired (and are still acquiring) in this social context.^15^ It is essential that the implicit knowledge transferred through this process becomes explicit.

Moreover, PIF does not cease when classes end; students continue to be socialised as their education and careers advance. Therefore, they not only need to become aware of their current socialisation processes but they also need to develop a reflective attitude to foster continuous awareness and reflection on these processes throughout their professional lives.^16^^-^^18^ A deep understanding and awareness of the organisational and social contexts of the medical system and how they influence individual and group functioning is therefore required.^17^^,^^19^

While searching for a teaching method to support PIF among medical students, we identified the Systemic Constellation (SC) method^20^^,^^21^ as a promising teaching method for enhancing learners' awareness of their implicit knowledge of their social context. This experiential learning method, which is intended for use in groups, stimulates reflection by the individual and the group. It entails zooming out from the level of the individual to the wider level of the social context. Through a group exercise, the social context as well as existing values, beliefs, habits and perspectives are visualised and rendered explicit. This process encourages active reflection on these values, beliefs, habits and perspectives and an exploration of individual differences. The method is already widely used in business settings for team development and leadership training^21^^,^^22^ and has also been applied within the education sector.^20^^,^^23^

In 2017, we began incorporating the SC method into the medical curriculum at our university. This training is offered as a component of a compulsory educational track on leadership development as well as in various elective courses on professionalisation, and in training programmes for teachers, interns and residents. To the best of our knowledge, the SC method has not been used within medical curricula to date. In this article, we reflect on the use of the SC method as a teaching method that supports PIF. Moreover, we share our experience of implementing this method in a medical education setting. In the following sections, we describe the SC method in more detail and illustrate its application in this setting with a concrete, practical example.

### The Systemic Constellation method

The SC method originates in systemic family constellations applied within clinical counselling settings.^24^ However, it has been developed further, and its application has been extended to other social systems, such as organisations and teams.^22^ The method is premised on a view that individuals intuitively know about the structures, relations and interdependencies of the components within the social systems to which they belong.^22^^,^^24^ This knowledge is often implicit, although it can be rendered explicit via the visualisation of a social system using a spatial arrangement of elements, which are relevant to the specific social context (a constellation). These elements comprise individuals or objects representing functions or roles within the social system (e.g., an intern) or groups/stakeholders (e.g., patients). They may also include more abstract concepts or societal aspects that are considered important in the social system (e.g., a value, procedure or concept like hierarchy). Accordingly, the social system and its interrelated components are made visible and tangible. Below, we provide details on the practical implementation of this method.

Tasked with inserting concrete or abstract elements of a social context into a physical space, and in relation to each other, learners must rely not only on facts or on established knowledge but they must also transform their implicit knowledge of the social structures into a visual and thus explicit form. By exploring this visualisation and reflecting on it together, learners become aware of their implicit knowledge regarding their social context and how it influences their own and others' functioning, wellbeing and learning. Moreover, by becoming aware of the acquired beliefs, values and information on the social context, students can evaluate how closely these beliefs and values are aligned with their own and the extent to which they wish to adopt these beliefs and values. Thus, the use of this method renders implicit social information explicit and imparts skills that enable learners to reflect actively on their PIF as an ongoing process.

A detailed description of the practical implementation of an SC is provided in a previous publication^22^ and at the university website (www.rug.nl/SCOPE). In brief, a SC is performed with a group led by a trained facilitator. The facilitator interviews one or more participants about a case. Together, they identify elements that are relevant to this case and visualise the social system, considering the other individuals present as representatives of the elements. These representatives do not have to play a part or receive instructions as in regular role-playing; they simply represent the assigned element. The facilitator explores the visualisation together with the participants. Once a point of saturation is reached, the constellation process is closed with collective reflections on the case and the sharing of insights.

### Example of the implementation of a systemic constellation

In a training programme aimed at developing collaboration skills as part of a track on professional development, students working together on a project perform a SC. One team is asked to name four elements that they encountered in the social context of their project, for example, their supervisor, patients, or other stakeholders. After the team members have identified the elements, the facilitator asks them to pick four representatives from among the other students present at the training. The four representatives stand up and occupy a self-chosen position in the room. Next, the facilitator invites the team members to position themselves within the constellation at the place that best represents their position at that moment. This process is followed by collective reflection involving the entire group.

When performing this exercise, students from the same team often expressed surprise upon finding that they occupied different positions within the constellation. These observations opened up conversations about their ideas and beliefs about roles and functions within the project and the social context of their project, which they were not previously aware of. The students reported that they learned that they held implicit beliefs about their social context and about their own position and role within that context. They also discovered that many of their implicit beliefs were shared by their peers, such as 'the doctor should always know' or 'if you work hard you will succeed'. Together, they gained insight into their socialisation process and its influence on their own functioning and that of others. Some students also discovered that from a rational perspective, they did not agree with some of their own implicit beliefs.

### Reflections on the implementation of the SC method

Our experiences confirm that the SC method can be implemented effectively in a medical educational setting. However, the presence of facilitators who are both acquainted with the medical education setting and trained in the SC method appears to be conducive to its success. Although students were often completely unfamiliar with the method, our experience showed that they were able to execute the facilitator's instructions following a brief introduction to the method. In the evaluations that we conducted relating to the use of the method within the curriculum, the majority of the students reported that they liked the training programme and that it gave them useful insights relating to their social context and professional development. Specifically, they appreciated the opportunity to visualise social dynamics and the new perspectives and insights that emerged from the exercise. We learned that the students were often not aware of the implicit and shared knowledge that they already had on their social context, which could be rendered explicit using the SC method and group reflection. Most students became aware of the influence of the social context on their thinking, functioning and wellbeing for the first time and collectively discussed this influence and the socialisation process as part of their PIF.

### In summary

In this article, we have proposed the use of the SC method as a potentially promising teaching method to guide PIF in medical students. Although its application in the medical education setting is new, it is widely used in other domains.^20^^-^^22^ While there are other methods that can support students in becoming aware of their unconscious beliefs, such as mask making^13^ and rich pictures,^25^^,^^26^ the SC method specifically addresses their unconscious beliefs about their social contexts. The visualisation of a social context is an important benefit associated with this method, as it enables individuals to capture an issue or context at a glance.^20^ This exercise enables all students to view the same external image of the social context as a representation of their own internal images but from different perspectives. Previous studies found that developing new perspectives on the social context was highly valued by participants in training programmes grounded in this method.^22^ Importantly, these programmes illuminated their implicit knowledge linked to their social context. This experience undergone collectively with other medical students was thus a shared experience, which allowed for personal reflection as well as collaborative learning, which are considered important elements in the PIF process.^4^

This article offers preliminary reflections on the feasibility and acceptability of applying the SC method in this setting. The next step would be to engage in further exploration of its effectiveness and to conduct a study to assess its outcomes, effects and contribution to the acquisition of professional identities. However, our experiences of using this method thus far indicate that it offers a promising approach for equipping medical professionals with an understanding of their socialisation process from an early stage. It can support the development of a reflective attitude among students and thereby facilitate their PIF.

### Acknowledgements

We would like to express our gratitude to the Hellinger Institute the Netherlands for their advice and inputs into our training programmes. We thank all the instructors and trainers who were involved in the development and execution of the training programmes using the Systemic Constellation method.

### Conflict of Interest

The authors declare that they have no conflict of interest.
